# Robust age, but limited sex, differences in mu-opioid receptors in the rat brain: relevance for reward and drug-seeking behaviors in juveniles

**DOI:** 10.1007/s00429-017-1498-8

**Published:** 2017-09-04

**Authors:** Caroline J. W. Smith, Aarane M. Ratnaseelan, Alexa H. Veenema

**Affiliations:** 1 Neurobiology of Social Behavior Laboratory, Department of Psychology, Boston College, 140 Commonwealth Ave, Chestnut Hill, MA 02467 USA; 2 Present Address: Department of Pediatrics, Massachusetts General Hospital for Children/Harvard Medical School, 114 16th Street, Charlestown, MA 02129 USA; 3 Present Address: Department of Psychology and Neuroscience Program, Michigan State University, 293 Farm Lane, East Lansing, MI 48824 USA

**Keywords:** µ-Opioid receptor, Sex differences, Age differences, Juvenile, Reward, Receptor autoradiography

## Abstract

In the brain, the µ-opioid receptor (MOR) is involved in reward-seeking behaviors and plays a pivotal role in the mediation of opioid use disorders. Furthermore, reward-seeking behaviors and susceptibility to opioid addiction are particularly evident during the juvenile period, with a higher incidence of opioid use in males and higher sensitivity to opioids in females. Despite these age and sex differences in MOR-mediated behaviors, little is known regarding potential age and sex differences in the expression of MORs in the brain. Here, we used receptor autoradiography to compare MOR binding densities between juvenile and adult male and female rats. Age differences were found in MOR binding density in 12 out of 33 brain regions analyzed, with 11 regions showing higher MOR binding density in juveniles than in adults. These include the lateral septum, as well as sub-regions of the bed nucleus of the stria terminalis, hippocampus, and thalamus. Sex differences in MOR binding density were observed in only two brain regions, namely, the lateral septum (higher in males) and the posterior cortical nucleus of the amygdala (higher in females). Overall, these findings provide an important foundation for the generation of hypotheses regarding differential functional roles of MOR activation in juveniles versus adults. Specifically, we discuss the possibility that higher MOR binding densities in juveniles may allow for higher MOR activation, which could facilitate behaviors that are heightened during the juvenile period, such as reward and drug-seeking behaviors.

## Introduction

The juvenile period, (synonymous with the early adolescent or peri-pubertal period in humans and spanning postnatal days 28–42 in rats; Spear 2000) is one during which individuals are particularly driven to seek rewards and to engage in drug-seeking and risk-taking behaviors (Spear 2000; Foulkes and Blakemore 2016; Casey et al. 2008; Compton and Volkow 2006). Moreover, juvenile rats are more likely to engage in social interactions with peers, and find these social interactions to be more rewarding than at younger or older ages (Spear 2000; Doremus-Fitzwater et al. 2010). Previous work in adolescent humans and juvenile rodents suggests an important role for the mu-opioid receptor (MOR) in the regulation of both drug seeking and social behaviors. For example, polymorphisms of the human MOR gene (*OPRM1*) are associated with alcohol misuse (Miranda et al. 2010) and differences in neural activation to reward and alcohol-related cues (Nees et al. 2017; Pieters et al. 2011). In rats, central MOR antagonism blocks the reinforcing properties of ethanol (Pautassi et al. 2011), reduces social play behavior (Trezza et al. 2011), and reduces social novelty preference (Smith et al. 2015). Based on these findings, we propose that the juvenile propensity to engage in social interaction, drug seeking, and risk-taking may be due to heightened MOR activation in the brain compared to younger and older ages. Yet, little is known regarding age differences in MOR expression in the brain. Although some studies have charted the pre-weaning development of MOR in the rat brain (Recht et al. 1985; Kornblum et al. 1987; Moon Edley and Herkenham 1984; Spain et al. 1985), a quantitative comparison of MOR binding densities between juveniles and adults is lacking. Therefore, our first aim was to determine MOR binding densities in the brains of juvenile and adult rats. We hypothesized that MOR binding density would be higher in juveniles compared to adults in brain regions involved in regulating reward and drug-seeking behaviors.

Several studies suggest that there are sex differences in reward-seeking behaviors and susceptibility to drug abuse. For example, boys are more likely to engage in reward and sensation seeking behaviors than girls (Steinberg et al. 2008; Romer and Hennessy 2007), while girls are more likely to consume alcohol than boys (Johnston et al. 2015). Moreover, men are more likely than women to engage in substance abuse (Lynch et al. 2002), while women become addicted to opiates more quickly following first use (Lex 1991; Roth et al. 2004). Similarly, female rats acquired heroin self-administration more quickly than their male counterparts, and subsequently, self-administered larger amounts of the drug (Lynch and Carroll 1999; Cicero et al. 2003). It is plausible that sex differences in MOR activation underlie sex differences in these behaviors. In support, PET scan studies revealed higher MOR binding in several brain regions of women compared to men (Zubieta et al. 1999). Likewise, higher MOR binding density was found in several brain regions in female rats compared to males, although these rats were gonadectomized (Vathy et al. 2003). However, it remains unknown whether sex differences are present in the intact rat brain and whether these sex differences emerge early in development. Therefore, our second aim was to compare MOR binding density between intact male and female rats at both juvenile and adult ages. Based on these previous findings in humans and gonadectomized adult rats (Zubieta et al. 1999; Vathy et al. 2003), we hypothesized that MOR binding density would be higher in females than in males.

## Methods

### Animals

Male and female Wistar rats were obtained from Charles River Laboratories (Raleigh, NC) at 22 or 56 days of age and housed under standard laboratory conditions (12-h light/dark cycle, lights on at 7:00 am, food and water available ad libitum, 22 °C, 60% humidity). Upon arrival at our facility, rats were housed in standard rat cages (26.7 × 48.3 × 20.3 cm). Twenty-two-day-old rats were housed in same-sex groups of 3–4 until brain collection for receptor autoradiography at 35 days of age (juvenile group). Fifty-six-day-old rats were housed in same-sex pairs until brain collection for receptor autoradiography at 84 days of age (adult group). All experiments were conducted in accordance with the NIH Guide to the Care and Use of Laboratory Animals and approved by the Boston College Institutional Animal Care and Use Committee (IACUC).

### Receptor autoradiography

Rats (juvenile males: *n* = 13; juvenile females: *n* = 13; adult males: *n* = 12; adult females: *n* = 12) were euthanized using CO_2_ inhalation and brains were removed, rapidly frozen in methylbutane on dry ice, and stored at −45 °C. Brains were cut on a cryostat into 16-µm coronal sections and mounted onto slides in eight adjacent series. Collection began at approximately 3.72 mm anterior to bregma and ended at approximately 8.52 mm posterior to bregma (Paxinos and Watson 2007). Sections were then frozen −45 °C until receptor autoradiography was performed. MOR autoradiography was conducted using the MOR-specific agonist [^3^H]D-Ala^2^-MePhe^4^-Gly-ol^5^ enkephalin (DAMGO; Perkin Elmer, Boston, MA) as tracer. In brief, slides were thawed and air-dried at room temperature, followed by pre-incubation for 30 min in 50 nM Tris–HCl (pH 7.4) containing 0.9% NaCl. The slides were then exposed to tracer buffer (4 nM [^3^H]D-Ala^2^-MePhe^4^-Gly-ol^5^ enkephalin and 50 mM Tris) for 60 min. Non-specific binding was assessed in adjacent brain sections, by incubation in tracer buffer with the addition of 1 µM of the MOR antagonist naloxone (Sigma-Aldrich, St. Louis, MO). All slides were then washed three times, for 5 min each, in ice-cold Tris–HCl, air-dried, and exposed to Biomax MR films (VWR International, Pittsburgh, PA) for 16 weeks. Brain sections of juvenile and adult male and female rats were processed together and balanced across incubation chambers and exposure to films.

### Image and data analysis

**Fig. 1:**
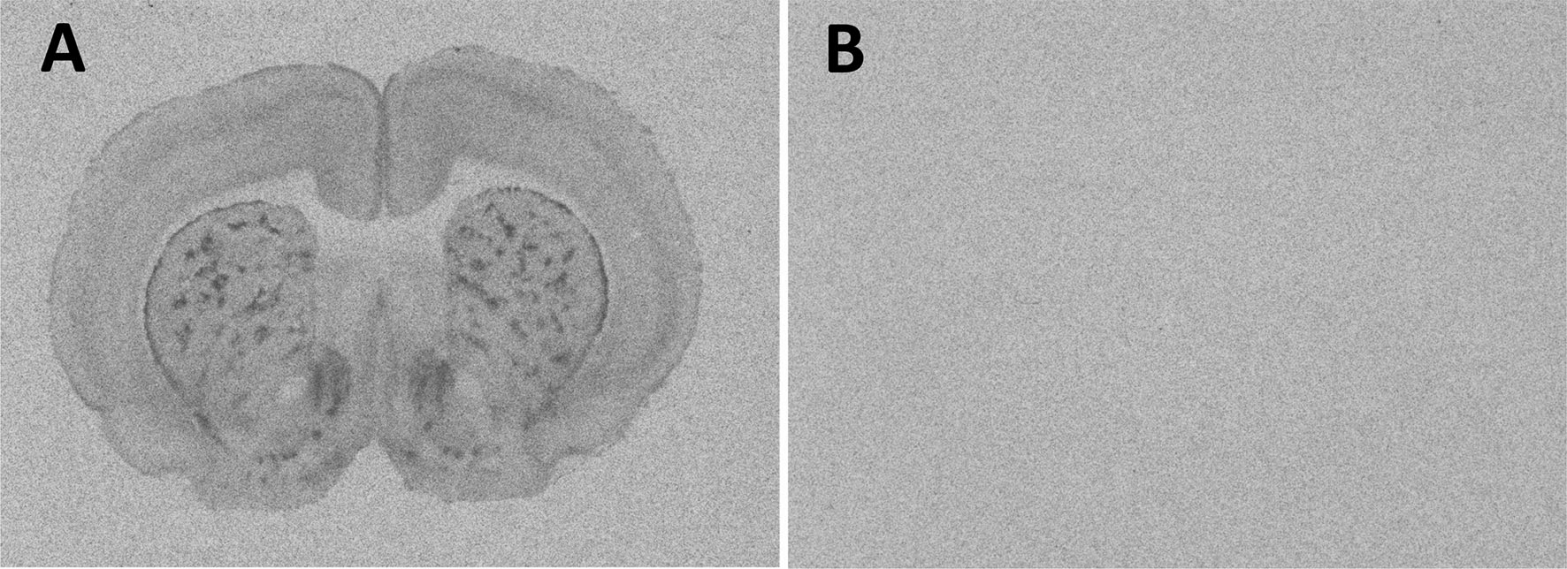
MOR binding in 16 μm coronal brain sections in the absence and presence of the selective MOR receptor antagonist naloxone. **a** Incubation with the radioligand [^3^H]D-Ala^2^-MePhe^4^-Gly-ol^5^ enkephalin yielded MOR binding in the striatum. **b** Incubation with the same radioligand and an excess of unlabeled naloxone yielded no binding, indicating that binding in **a** is specific to the MOR

**Fig. 2:**
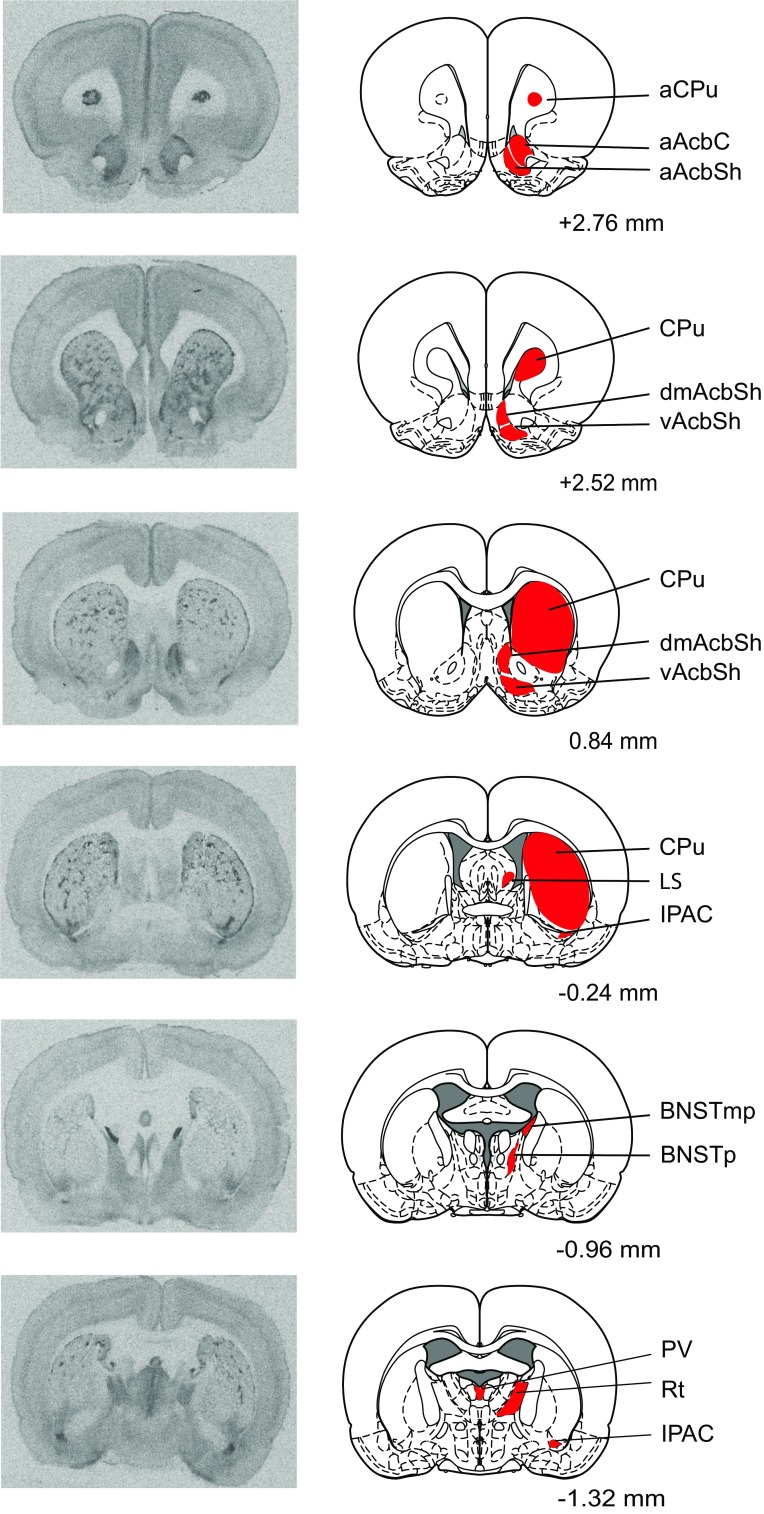

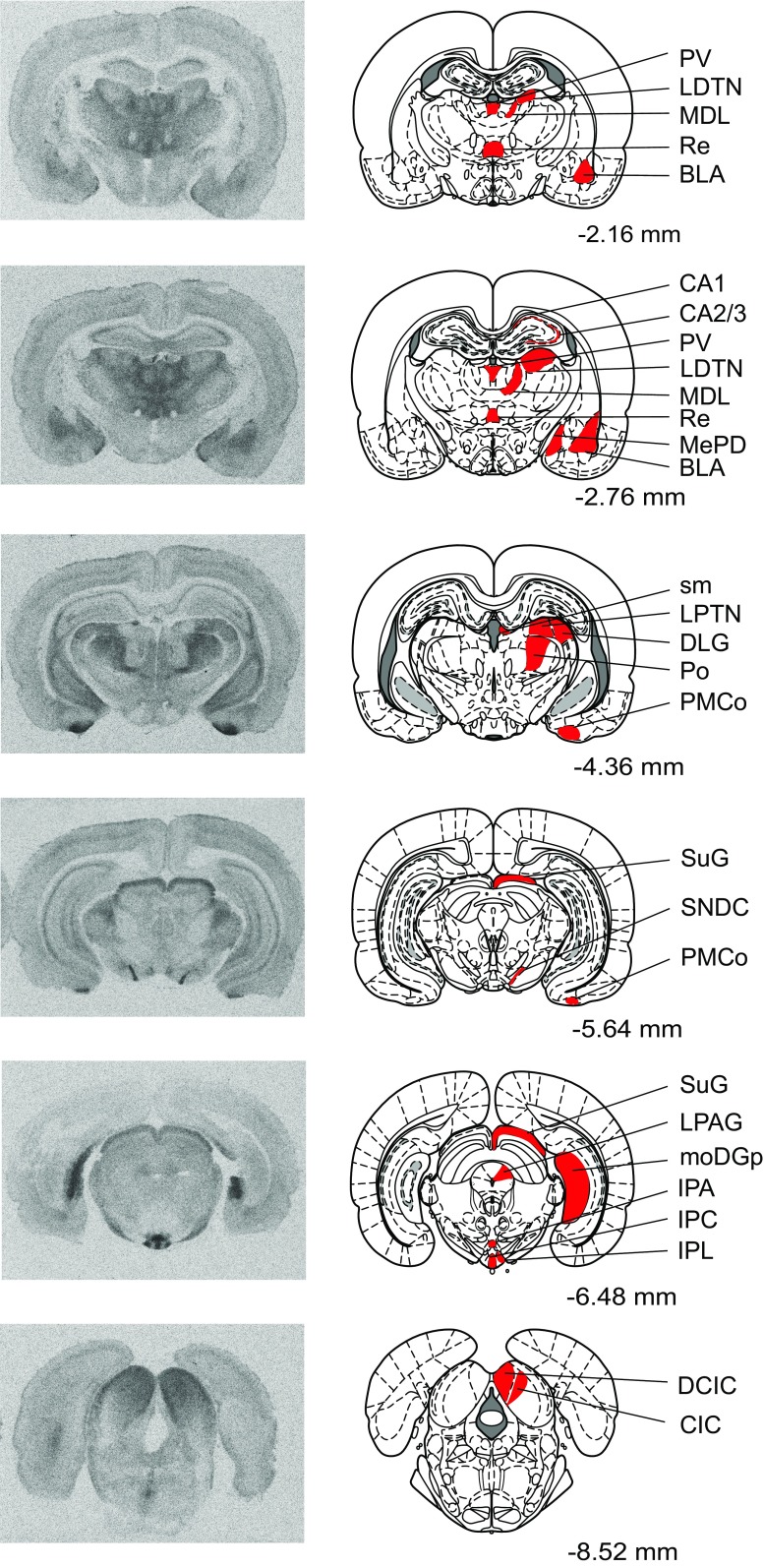
Representative autoradiograms of MOR binding in coronal rat brain sections. Brain regions in which MOR binding was measured are highlighted in* red*. Distances are measured in millimeters from bregma, according to Paxinos and Watson (2007). Note that while many brain regions are analyzed across multiple bregma distances, regions are highlighted in the most representative atlas images only

Autoradiography films were digitized using a Northern Light Illuminator (InterFocus Imaging, Cambridge, UK) and optical densities of MOR binding were measured in coronal sections using ImageJ (NIH, http://imagej.nih.gov/ij/). The data were converted to dpm/mg tissue (disintegrations per minute/milligram tissue) using a [^3^H] standard microscale (American Radiolabeled Chemicals Inc., St. Louis, MO). Because non-specific binding was undetectable (Fig. 1), film background values were subtracted from total binding values to yield specific binding values. Binding densities were calculated by taking the mean of bilateral measurements in a fixed number of sections per region of interest per rat. The total number of measurements depended on the size of the region of interest and ranged from 4 to 13. MOR binding density was measured in a total of 33 brain regions (see Fig. 2 for receptor autoradiograms and schematic diagrams indicating the brain regions in which MOR binding was quantified). All abbreviations of brain regions are in accordance with Paxinos and Watson (2007), except for the nucleus accumbens core and nucleus accumbens shell, where we added the subdivisions anterior core (aAcbC), anterior shell (aAcbSh), dorsomedial shell (dmAcbSh) and ventral shell (vAcbSh) to delineate the separate areas analyzed, as well as for the laterodorsal thalamic nucleus where we used the abbreviation LDTN to refer to the dorsomedial and ventrolateral parts of the nucleus combined, the lateral posterior thalamic nucleus where we used the abbreviation LPTN to refer to the mediorostral and laterorostral parts combined, and the molecular layer of the dentate gyrus where we used the abbreviation moDGp to refer to the more posterior part of the region.

### Statistics

For all statistical analysis, PASW/SPSS Statistics (Version 22.0) was used. A one-way ANOVA followed by Bonferroni post-hoc testing was used to compare MOR binding density across all brain regions analyzed and collapsed across age and sex. Two-way ANOVAs were used to test for age and sex differences in MOR binding density in each brain region. The false discovery rate (FDR) procedure (Hochberg and Benjamini 1990) was used to correct for multiple comparisons (age, sex, and interaction). This resulted in an FDR *α* < 0.0130 (based on 99 comparisons). Significant interaction effects were followed by Bonferroni post hoc tests (reflecting *t* tests pre-adjusted for multiple comparisons) to examine differences among groups. Significant age or sex effects were followed by Cohen’s *D* to calculate the effect size of age differences (overall and separately for males and females) and of sex differences (overall and separately for juveniles and adults). A subsequent independent samples *t* test was run to determine whether the effect size of age differences was different between males and females for all brain regions. Significance for independent samples *t* tests was set at *p* < 0.05.

## Results

### Brain region-specific patterns of MOR binding density

**Fig. 3:**
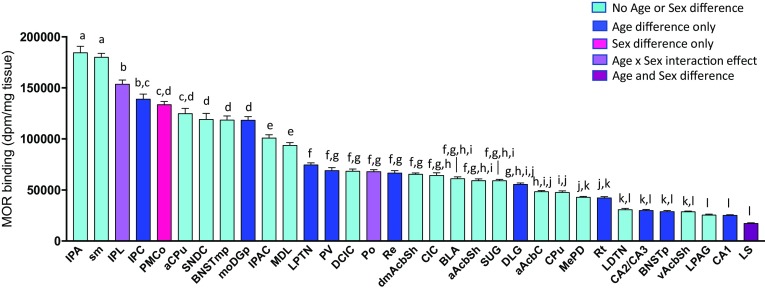
MOR binding density levels across brain regions. Brain regions in which MOR binding density was analyzed, are organized from highest (*left*) to lowest (*right*) MOR binding density. MOR binding densities are collapsed for both age and sex per brain region. Data represent mean + SEM; *bars* without* letters* in common differ significantly (*p* < 0.05) based on one-way ANOVA (*brain region*) followed by Bonferroni post hoc comparisons. *Color coding* indicates brain regions in which age, sex, age × sex, or no effects for MOR binding density were observed

MOR binding density varied greatly by brain region [*F*_(32,1527)_ = 260.8; *p* < 0.001; Fig. 3], with an approximate tenfold difference between the highest and lowest MOR binding density within the 33 regions that were analyzed. Binding density was highest in the apical subnucleus of the interpeduncular nucleus (IPA) and stria medullaris of the thalamus (sm) and lowest in the lateral periaqueductal gray (LPG), CA1 region of the hippocampus (CA1), and lateral septum (LS) (Fig. 3). Notably, MOR binding densities did not predict where age, sex, and age × sex interaction effects were found.

### Age differences in MOR binding density

**Fig. 4:**
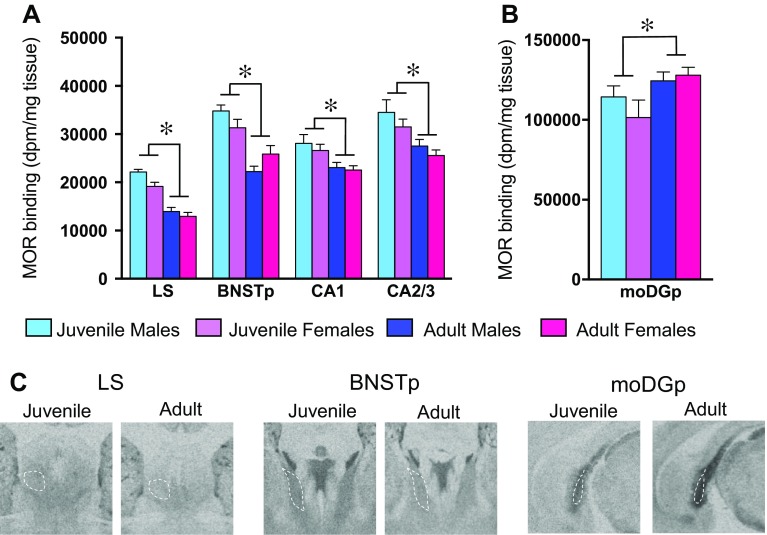
Age differences in MOR binding density in the telencephalon. Brain regions in which MOR binding density is higher (**a**) or lower (**b**) in juveniles as compared to adults within the telencephalon. Representative autoradiograms of age differences in MOR binding density in the LS, BNSTp, and moDGp (**c**). *Bars* in **a**, **b** indicate mean + SEM; two-way ANOVA (age × sex) with FDR correction for multiple comparisons: *FDR *α* < 0.013

**Fig. 5:**
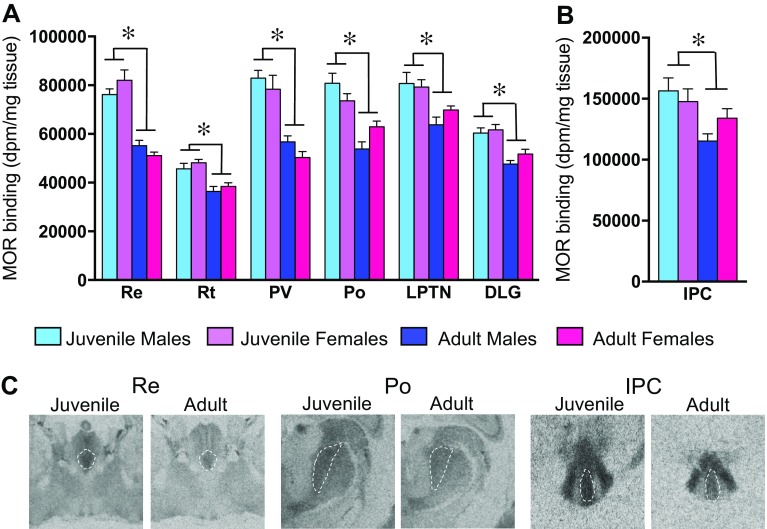
Age differences in MOR binding density in the diencephalon and mesencephalon. Brain regions in which MOR binding density is higher in juveniles as compared to adults within the diencephalon (**a**) and mesencephalon (**b**). Representative autoradiograms of age differences in MOR binding density in the Re, Po, and IPC (**c**). *Bars* in **a**, **b** indicate mean + SEM; two-way ANOVA (age × sex) with FDR correction for multiple comparisons: *FDR *α* < 0.013

**Fig. 6:**
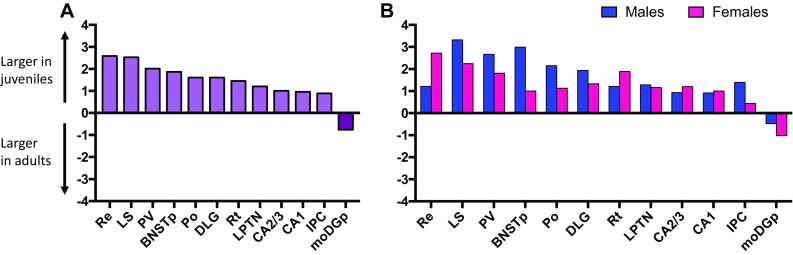
Cohen’s *D* effect size measurements for all significant age differences in MOR binding density, collapsed across sexes (**a**) and separately in males and females (**b**)

Age differences in MOR binding density were found in 12 of the 33 brain regions analyzed (see Table 1 for complete statistics). MOR binding density was higher in juveniles compared to adults in 11 brain regions: 4 telencephalic brain regions [LS, the CA1 and CA2/3 sub-regions of the hippocampus, and the posterior bed nucleus of the stria terminalis (BNSTp; Fig. 4a)], 6 diencephalic brain regions [the dorsal lateral geniculate nucleus (DLG), the LPTN, the posterior thalamic nuclear group (Po), the paraventricular thalamic nucleus (PV), the reticular thalamic nucleus (Rt), and the nucleus reuniens (Re); Fig. 5a] and the caudal subnucleus of the interpeduncular nucleus in the mesencephalon (IPC; Fig. 5b). MOR binding density was higher in adults than juveniles only in the moDGp (Fig. 4b). Finally, the effect sizes of age differences were similar between the sexes [*t*_(1,22)_ = −1.21; *p* = 0.24; Fig. 6a, b].

**Table 1:** Statistical details of age, sex, and interaction effects on MOR binding densities in the rat brain

	Direction	Age effect	Sex effect	Interaction effect
Telencephalon				
Striatal areas				
aCPu		*F*_(1,40)_ = 0.34; *p* = 0.56	*F*_(1,40)_ = 3.52; *p* = 0.07	*F*_(1,40)_ = 0.69; *p* = 0.41
CPu		*F*_(1,46)_ = 0.23; *p* = 0.63	*F*_(1,46)_ = 0.48; *p* = 0.49	*F*_(1,46)_ = 0.03; *p* = 0.86
aAcbC		*F*_(1,46)_ = 0.30; *p* = 0.59	*F*_(1,46)_ = 1.73; *p* = 0.19	*F*_(1,46)_ = 0.22; *p* = 0.64
aAcbSh		*F*_(1,46)_ = 0.19; *p* = 0.66	*F*_(1,46)_ = 1.16; *p* = 0.29	*F*_(1,46)_ = 4.01; *p* = 0.05
dmAcbSh		*F*_(1,46)_ = 0.14; *p* = 0.71	*F*_(1,46)_ = 1.24; *p* = 0.27	*F*_(1,46)_ = 0.68; *p* = 0.41
vAcbSh		*F*_(1,46)_ = 4.59; *p* = 0.04	*F*_(1,46)_ = 0.10; *p* = 0.75	*F*_(1,46)_ = 0.16; *p* = 0.69
Septal areas				
LS	Higher in juveniles and males	***F***_**(1,42)**_ **=** **86.2;** ***p*** **<** **0.001**	***F***_**(1,42)**_ **=** **6.73;** ***p*** **=** **0.013**	*F*_(1,42)_ = 1.63; *p* = 0.21
Bed nucleus of the stria terminalis areas				
BNSTpm		*F*_(1,45)_ = 4.32; *p* = 0.04	*F*_(1,45)_ = 0.14; *p* = 0.71	*F*_(1,45)_ = 1.54; *p* = 0.22
BNSTp	Higher in juveniles	***F***_**(1,41)**_ **=** **38.29;** ***p*** **<** **0.001**	*F*_(1,41)_ = 0.00; *p* = 0.95	*F*_(1,41)_ = 5.95; *p* = 0.02
Amygdala areas				
IPAC		*F*_(1,46)_ = 0.46; *p* = 0.50	*F*_(1,46)_ = 1.12; *p* = 0.30	*F*_(1,46)_ = 0.05; *p* = 0.83
MePD		*F*_(1,46)_ = 0.93; *p* = 0.34	*F*_(1,46)_ = 0.26; *p* = 0.61	*F*_(1,46)_ = 2.76; *p* = 0.10
BLA		*F*_(1,46)_ = 5.84; *p* = 0.02	*F*_(1,46)_ = 0.04; *p* = 0.83	*F*_(1,46)_ = 0.06; *p* = 0.80
PMCO	Higher in females	*F*_(1,46)_ = 0.38; *p* = 0.54	***F***_**(1,46)**_ **=** **9.56;** ***p*** **<** **0.005**	*F*_(1,46)_ = 6.60; *p* = 0.01
Hippocampal areas				
CA1	Higher in juveniles	***F***_**(1,46)**_ **=** **11.0;** ***p*** **<** **0.002**	*F*_(1,46)_ = 0.53; *p* = 0.47	*F*_(1,46)_ = 0.13; *p* = 0.72
CA2/3	Higher in juveniles	***F***_**(1,46)**_ **=** **12.5;** ***p*** **<** **0.001**	*F*_(1,46)_ = 1.85; *p* = 0.18	*F*_(1,46)_ = 0.08; *p* = 0.77
MoDGp	Higher in adults	***F***_**(1,40)**_ **=** **7.02;** ***p*** **=** **0.012**	*F*_(1,40)_ = 0.46; *p* = 0.50	*F*_(1,40)_ = 1.42; *p* = 0.24
Diencephalon				
Thalamic areas				
DLG	Higher in juveniles	***F***_**(1,46)**_ **=** **32.1;** ***p*** **<** **0.001**	*F*_(1,46)_ = 1.83; *p* = 0.18	*F*_(1,46)_ = 0.49; *p* = 0.49
LDTN		*F*_(1,40)_ = 0.18; *p* = 0.67	*F*_(1,40)_ = 4.02; *p* = 0.05	*F*_(1,40)_ = 3.16; *p* = 0.08
LPTN	Higher in juveniles	***F***_**(1,38)**_ **=** **12.8;** ***p*** **<** **0.001**	*F*_(1,38)_ = 0.39; *p* = 0.54	*F*_(1,38)_ = 1.05; *p* = 0.31
MDL		*F*_(1,40)_ = 0.09; *p* = 0.77	*F*_(1,40)_ = 0.25; *p* = 0.62	*F*_(1,40)_ = 0.65; *p* = 0.42
Po	Higher in juveniles	***F***_**(1,45)**_ **=** **35.1;** ***p*** **<** **0.001**	*F*_(1,45)_ = 0.09; *p* = 0.76	***F***_**(1,45)**_ **=** **6.63;** ***p*** **=** **0.01**
PV	Higher in juveniles	***F***_**(1,40)**_ **=** **41.4;** ***p*** **<** **0.001**	*F*_(1,40)_ = 1.70; *p* = 0.20	*F*_(1,40)_ = 0.05; *p* = 0.82
Re	Higher in juveniles	***F***_**(1,46)**_ **=** **84.0;** ***p*** **<** **0.001**	*F*_(1,46)_ = 0.10; *p* = 0.75	*F*_(1,46)_ = 3.07; *p* = 0.09
Rt	Higher in juveniles	***F***_**(1,46)**_ **=** **26.2;** ***p*** **<** **0.001**	*F*_(1,46)_ = 1.52; *p* = 0.22	*F*_(1,46)_ = 0.02; *p* = 0.89
sm		*F*_(1,46)_ = 4.64; *p* = 0.04	*F*_(1,46)_ = 0.23; *p* = 0.63	*F*_(1,46)_ = 0.38; *p* = 0.54
Mesencephalon				
Tectal areas				
SuG		*F*_(1,46)_ = 4.39; *p* = 0.04	*F*_(1,46)_ = 2.32; *p* = 0.13	*F*_(1,46)_ = 0.37; *p* = 0.54
CIC		*F*_(1,36)_ = 2.72; *p* = 0.11	*F*_(1,36)_ = 0.40; *p* = 0.53	*F*_(1,36)_ = 0.10; *p* = 0.75
DCIC		*F*_(1,46)_ = 4.66; *p* = 0.04	*F*_(1,46)_ = 1.83; *p* = 0.18	*F*_(1,46)_ = 0.49; *p* = 0.49
Tegmental areas				
LPAG		*F*_(1,39)_ = 4.56; *p* = 0.04	*F*_(1,39)_ = 0.15; *p* = 0.70	*F*_(1,39)_ = 2.90; *p* = 0.10
SNCD		*F*_(1,44)_ = 3.97; *p* = 0.05	*F*_(1,44)_ = 0.09; *p* = 0.77	*F*_(1,44)_ = 0.12; *p* = 0.73
IPA		*F*_(1,42)_ = 0.57; *p* = 0.45	*F*_(1,42)_ = 0.03; *p* = 0.87	*F*_(1,42)_ = 3.56; *p* = 0.07
IPC	Higher in juveniles	***F***_**(1,42)**_ **=** **9.13;** ***p*** **<** **0.005**	*F*_(1,42)_ = 0.30; *p* = 0.59	*F*_(1,42)_ = 2.27; *p* = 0.14
IPL	Higher in juvenile males	*F*_(1,42)_ = 5.26; *p* = 0.03	*F*_(1,42)_ = 0.85; *p* = 0.36	***F***_**(1,42)**_ **=** **10.1;** ***p*** **<** **0.005**

Significant effects (two-way ANOVA with FDR correction: *p* < 0.0130) are bolded

### Sex differences in MOR binding density

**Fig. 7:**
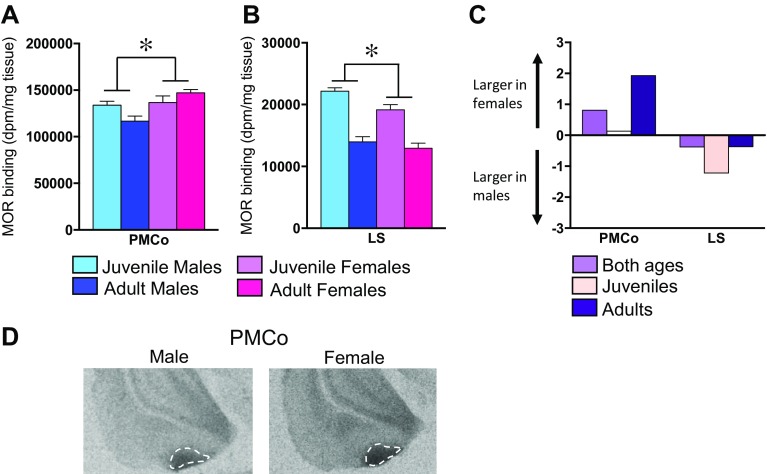
Sex differences in MOR binding density. MOR binding density is higher in females than in males in the PMCo (**a**) and higher in males than in females in the LS (**b**). Cohen’s *D* effect size measurements for significant sex differences collapsed across age and separately in juveniles and adults (**c**). Representative autoradiograms of MOR binding in the PMCo in an adult male and an adult female (**d**). *Bars* in **a**, **b** indicate mean + SEM; two-way ANOVA (age × sex) with FDR correction for multiple comparisons: *FDR *α* < 0.013

Sex differences in MOR binding density were observed in 2 of the 33 brain regions analyzed (see Table 1 for complete statistics). In detail, MOR binding density was higher in females than in males in the posteromedial cortical amygdaloid nucleus (PMCo; Fig. 7a) with the effect size being larger in adults than in juveniles (Fig. 7c). In contrast, MOR binding density was higher in males than in females in the LS (Fig. 7b) with the effect size being larger in juveniles than in adults (Fig. 7c).

### Age × sex interactions in MOR binding density

**Fig. 8:**
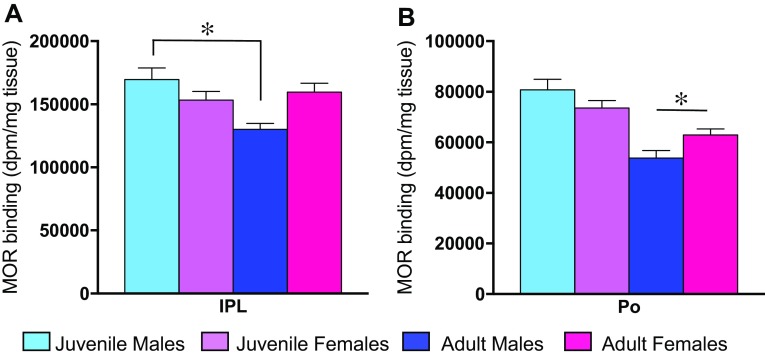
Age × sex interaction effects for MOR binding density. In the IPL, MOR binding density is significantly higher in juvenile males as compared to adult males (**a**). In the Po, MOR binding density is higher in adult females as compared to adult males (**b**). *Bars* indicate mean + SEM; two-way ANOVA (age × sex) with FDR correction for multiple comparisons (*α* < 0.013) followed by Bonferroni post hoc tests (*p < 0.05)

Significant age × sex interaction effects on MOR binding density were found in two brain regions (see Table 1 for complete statistics). In detail, in the lateral subnucleus of the interpeduncular nucleus (IPL), MOR binding density was higher in juveniles compared to adults in males (*p* < 0.001), but not in females (*p* = 0.534; Fig. 8a). In the Po, adult males had significantly lower MOR binding density than adult females (*p* < 0.05), while there was no sex difference in juveniles (*p* = 0.112; Fig. 8b).

### Similar MOR binding density between the ages and sexes

Despite robust MOR binding, no age or sex differences were found in 19 of the 33 brain regions analyzed, including 10 telencephalic regions, 3 diencephalic regions, and 6 mesencephalic regions (see Table 1 for statistics and list of brain regions).

## Discussion

Using receptor autoradiography, we show that MOR binding is found in numerous regions throughout the rat brain and that there is considerable variation in the density of MOR binding across brain regions. Importantly, this is the first study to quantitatively compare MOR binding density in the brain between juvenile and adult male and female rats. Age differences in MOR binding density were found in 12 out of 33 brain regions analyzed. All but one of these age differences demonstrate higher MOR binding density in juveniles compared to adults, and were predominantly seen in thalamic sub-regions. Interestingly, sex differences in MOR binding density were found in only 2 of the 33 brain regions assessed. Taken together, these findings demonstrate that MOR binding density varies considerably with age, but that sex may not be an important determining variable of MOR binding density. The higher MOR binding density in juveniles than adults may allow for enhanced MOR activation. This, in turn, may be required for the regulation of juvenile-typical behaviors. This hypothesis is discussed further below.

### MOR binding density across the rat forebrain and midbrain: species comparisons and functional relevance

MOR binding density varied substantially across regions of the rat brain, independent of age and sex. In general, MOR binding was observed in the same brain regions as previously reported in the adult male rat using MOR autoradiography (Mansour et al. 1986, 1987; Temple and Zukin 1987; McLean et al. 1986; Mansour et al. 1994) or MOR immunohistochemistry (Ding et al. 1996), with the latter suggesting that MOR binding density resembles MOR protein expression. Moreover, the patterns of MOR binding density in the rat brain are largely consistent with those reported in other mammalian species (Daunais et al. 2001; Hurd and Herkenham 1993; Voorn et al. 1996; Ragen et al. 2015a, b). In particular, dense MOR binding in striatal and amygdala sub-regions has been observed in species ranging from rats, voles, and guinea pigs to non-human primates and humans (Daunais et al. 2001; Hurd and Herkenham 1993; Voorn et al. 1996; Ragen et al. 2015a, b; Inoue et al. 2013; Resendez et al. 2013; Sharif and Hughes 1989). Furthermore, the notable absence of MOR binding in the central nucleus of the amygdala observed in rats is consistent with reports in titi monkeys and macaques (Daunais et al. 2001; Ragen et al. 2015a, b). Even patterns of MOR binding density within individual brain regions appear to be consistent across species in some cases. For example, here we show that in rats MOR binding density is higher in the anterior than in medial/posterior portions of the caudate putamen (CPu) and MOR binding density is lower in the ventral part compared to the dorsomedial and anterior parts of the nucleus accumbens shell. This pattern is in line with MOR binding density in the CPu in prairie voles, meadow voles and macaques (Resendez et al. 2013; Daunais et al. 2001) and in the nucleus accumbens shell in prairie and meadow voles (Resendez et al. 2013). Given the assumption that differences in MOR binding density may reflect differences in MOR activation, the consistency of these MOR binding density patterns across species could indicate that the functions of MORs in these sub-regions are conserved. Indeed, the neural circuitry underlying pleasure and reward (including the nucleus accumbens and CPu) is highly evolutionarily conserved, and evidence for the involvement of MORs in pleasure and reward can be found in all of the above-mentioned species (Berridge and Kringelbach; 2015; Resendez et al. 2013; Trezza et al. 2011; Hsu et al. 2013; Ragen et al. 2015b; Barr et al. 2010).

### Age differences in MOR binding density: role of synaptic pruning during development?

Age differences in MOR binding density were observed in 12 out of 33 brain regions analyzed, including the lateral septum, as well as sub-regions of the hippocampus, BNST, thalamus, and interpeduncular nucleus. Importantly, the direction of these age differences was largely uniform with denser MOR binding in juveniles than adults. Moreover, these age differences were found in both sexes. Previous studies in rats have shown that, in many brain regions, MOR binding density is highest around postnatal day 12 compared to earlier ages and to adulthood (Recht et al. 1985; Kornblum et al. 1987; Moon Edley and Herkenham 1984; Spain et al. 1985). The decline in MOR binding density after postnatal day 12 has been suggested to be the result of increased synaptic pruning in the third and fourth weeks of postnatal life in the rat (Kornblum et al. 1987). However, synaptic elimination is a developmental process that continues well into the pubertal period (Andersen et al. 2000; Huttenlocher and Dabholkar 1997; Geröcs et al. 1986). Therefore, it is plausible that the brain regions in which we observed higher MOR binding densities in juvenile than in adult rats are those in which synaptic pruning is not complete until later in development. If so, one might expect to see a similar decline in the binding density of other types of receptors in the same brain regions. Yet, oxytocin receptor binding density in the BNSTp has been found to be higher in adult compared to juvenile rats (Smith et al. 2017). Furthermore, dopamine D1, D2, and D4 receptor binding has been shown to be lower in adult compared to juvenile rats in the nucleus accumbens (Tarazi and Baldessarini 2000), while we did not observe an age difference in MOR binding density in this region. Therefore, if pruning is causing a decline in receptors, these findings suggest that it may be specific to synapses expressing only certain types of receptors and not others.

### Age differences in MOR binding density: possible functional implications for reward and drug seeking behaviors

Irrespective of the underlying cause of the age-dependent decline in MOR binding density, it is likely that higher MOR binding density in juveniles allows for higher MOR activation, which may have relevance to the facilitation of juvenile-specific behaviors. One region of particular interest is the lateral septum, because it shows the most robust age difference in MOR binding density, is reciprocally connected to the mesolimbic reward system (Swanson 1982), and it is involved in social play (Veenema et al. 2013; Bredewold et al. 2014, 2015), a highly rewarding and juvenile-specific behavior (Vanderschuren et al. 2016). Surprisingly, the role of MORs in the lateral septum in the regulation of social or non-social rewarding behavior has not been studied. However, MORs in the lateral septum have been implicated in the regulation of anxiety. Specifically, MOR activation increases anxiety-related behavior in adult mice (Le Merrer et al. 2006). This corresponds to the overall role of the lateral septum, as activation of this region results in anxiogenic effects (Anthony et al. 2014; Veening et al. 2009). It would, therefore, be interesting to determine whether higher MOR binding density in the lateral septum of juvenile versus adult rats has implications for the age-specific regulation of anxiety and rewarding social behaviors.

Age differences in MOR binding density were particularly evident in the thalamus, with six out of nine sub-regions showing higher MOR binding density in juvenile compared to adult rats. Although the functional role of MORs in these thalamic sub-regions is unknown, interesting and testable hypotheses can be generated based on the function of each of these sub-regions. For example, the nucleus reuniens receives input from the prefrontal cortex and relays it to the hippocampus (Ito et al. 2015; Hallock et al. 2016). Disruption of this pathway by blockade of the reuniens impairs spatial navigation, learning, and memory (Ito et al. 2015; Davoodi et al. 2009). Because MOR activation has an overall inhibitory effect on the thalamus (Brunton and Charpak 1998; Nakahama et al. 1981; Benoist et al. 1986), it is possible that denser MOR binding in the nucleus reuniens allows for higher MOR activation, which, in turn may mediate a greater inhibition of this circuit in juveniles than adults. Furthermore, the paraventricular nucleus of the thalamus mediates the aversive effects of opiate withdrawal through connections with the nucleus accumbens (Zhu et al. 2016). Because juveniles are less sensitive to the aversive effects of withdrawal than adults (Doremus-Fitzwater and Spear 2007; Hodgson et al. 2010) it is plausible that denser MOR binding allows for higher MOR activation in the paraventricular thalamic nucleus, which in turn may inhibit signaling in this pathway, resulting in reduced withdrawal symptoms in juveniles. These hypotheses will need to be tested in future studies.

Finally, age differences in MOR binding density were found in the caudal sub-region (higher in juveniles of both sexes) and in the lateral sub-region (higher in juveniles, but only in males) of the interpeduncular nucleus. This nucleus is densely interconnected with the lateral habenula (Sutherland 1982). This habenulo-interpeduncular pathway exerts a chronic inhibitory influence over the mesolimbic reward pathway (Nishikawa et al. 1986). In fact, it has been suggested that these two pathways jointly regulate the rewarding properties of drugs (Ellison 1994). Given the increased susceptibility to drug seeking behavior during the juvenile period (Spear 2000), it would be of interest to determine whether higher MOR binding density in the interpeduncular nucleus leads to higher susceptibility to MOR activation within the habenulo-interpeduncular pathway in juveniles versus adults. This, in turn, might result in higher susceptibility to drug-seeking behaviors in juveniles.

### Sex differences in MOR binding density

Sex differences in MOR binding density were found in the lateral septum (higher in males) and posterior cortical nucleus of the amygdala (higher in females). These sex differences were already present in juveniles, suggesting a pre-pubertal age of onset. Interestingly, both brain regions have sexually dimorphic features. The intermediate lateral septum, in which MOR binding was measured, contains more cells in females than in males (Segovia et al. 2009), while the posterior cortical nucleus of the amygdala contains more cells in males than in females (Vinader-Caerols et al. 1998). Thus, the direction of the sex difference in cell number is opposite to the direction of the sex difference in MOR binding density in both regions. Interestingly, the sex difference in posterior cortical nucleus volume and cell number is present prior to puberty (Akhmadeev and Kalimullina 2014) and in gonadectomized rats (Vathy et al. 2003), suggesting that this sex difference does not depend on circulating gonadal hormones.

Further research is required to determine the functional implications of the sex differences in MOR binding density in these two brain regions. The lateral septum is implicated in the regulation of emotional, motivational, and social behaviors (Sheehan et al. 2004). As such, higher MOR binding density in the lateral septum of males versus females may allow for higher MOR activation in males, which, in turn, may be involved in the regulation of any of these behaviors in a sex-specific way. The posterior cortical nucleus of the amygdala receives olfactory information from the main and accessory olfactory bulbs (Scalia and Winans 1975; Kevetter and Winans 1981), as well as the medial amygdala (DiBenedicitis et al. 2014) and mediates olfactory-guided social behaviors such as sexual behavior (Maras and Petrulis 2008). It would, therefore, be interesting to determine whether MORs in this brain region are involved in the sex-specific regulation of olfactory-guided social behaviors.

In contrast to our hypothesis, MOR binding density was similar between males and females in the vast majority of brain regions analyzed. A limitation of the current study is that we did not measure the effect of estrous phase on MOR binding density. However, MOR binding density variability (as interpreted by the average standard deviation of binding density in each brain region) was no greater in females than in males, suggesting that it is unlikely that estrous phase had a large impact on the absence of sex differences in MOR binding density. This suggests that the sex differences in opioid sensitivity and response in rats (Lynch and Carroll 1999; Cicero et al. 2003) are less likely to be due to sex differences in MOR binding. However, it is possible that sex differences occur in the downstream signaling pathways of the MOR. In support, estrogens have been shown to inhibit MOR-mediated signaling via a protein kinase A pathway (Wagner et al. 1998). Further work is needed to determine whether other aspects of the MOR system show sex differences and if so, whether these underlie the observed sex differences in MOR-mediated addictive behaviors.

## Conclusion

Our results demonstrate that age differences in MOR binding density in the rat brain are highly prevalent, while sex differences are not. We find numerous brain regions in which MOR binding density is higher in juveniles compared to adults, providing a potential mechanism for heightened MOR activation in the juvenile period that might be linked to higher engagement in reward and drug-seeking behaviors. Overall, the observation of robust age differences in MOR binding density provides an important first step in generating and testing hypotheses concerning the involvement of MORs in heightened expression of reward and drug-seeking behaviors in juveniles.

## Abbreviations

aAcbC: Anterior nucleus accumbens core
aAcbSh: Anterior nucleus accumbens shell
aCPu: Anterior caudate putamen
BLA: Basolateral amygdala
BNSTmp: Bed nucleus of the stria terminalis, posteromedial part
BNSTp: Bed nucleus of the stria terminalis, posterior part
CA1: CA1 layer of the hippocampus
CA2/3: CA2/3 layers of the hippocampus
CIC: Central nucleus of the inferior colliculus
CPu: Caudate putamen
DCIC: Dorsal cortex of the inferior colliculus
DLG: Dorsal lateral geniculate nucleus
dmAcbSh: Dorsomedial nucleus accumbens shell
IPA: Apical subnucleus of the interpeduncular nucleus
IPAC: Interstitial nucleus of the posterior limb of the anterior commissure
IPC: Caudal subnucleus of the interpeduncular nucleus
IPL: Lateral subnucleus of the interpeduncular nucleus
LDTN: Laterodorsal thalamic nucleus
LPA: Lateral periaqueductal gray
LPTN: Lateroposterior thalamic nucleus
LS: Lateral septum
MDT: Mediodorsal thalamus, lateral part
MePD: Medial amygdala, posterodorsal part
moDGp: Molecular layer of the dentate gyrus, posterior part
MOR: µ-opioid receptor
PMCo: Posteromedial cortical amygdaloid nucleus
Po: Posterior thalamic nuclear group
PV: Paraventricular thalamic nucleus
Re: Reuniens nucleus of the thalamus
Rt: Reticular nucleus of the thalamus
sm: Stria medullaris of the thalamus
SNCD: Substantia nigra, dorsal tier, compact part
SUG: Superficial gray layer of the superior colliculus
vAcbSh: Ventral nucleus accumbens shell
